# Towards Human Activity Recognition: A Hierarchical Feature Selection Framework

**DOI:** 10.3390/s18113629

**Published:** 2018-10-25

**Authors:** Aiguo Wang, Guilin Chen, Xi Wu, Li Liu, Ning An, Chih-Yung Chang

**Affiliations:** 1 School of Computer and Information, Hefei University of Technology, Hefei 230601, China; wangaiguo2546@163.com (A.W.); wuxi@hfut.edu.cn (X.W.); ning.an@hfut.edu.cn (N.A.); 2 School of Computer and Information Engineering, Chuzhou University, Chuzhou 239000, China; 3 School of Big Data and Software, Chongqing University, Chongqing 401331, China; dcsliuli@cqu.edu.cn; 4 Department of Computer Science and Information Engineering, Tamkang University, Tapei 25137, Taiwan; cychang@mail.tku.edu.tw

**Keywords:** activity recognition, hierarchical model, feature selection, information infusion

## Abstract

The inherent complexity of human physical activities makes it difficult to accurately recognize activities with wearable sensors. To this end, this paper proposes a hierarchical activity recognition framework and two different feature selection methods to improve the recognition performance. Specifically, according to the characteristics of human activities, predefined activities of interest are organized into a hierarchical tree structure, where each internal node represents different groups of activities and each leaf node represents a specific activity label. Then, the proposed feature selection methods are appropriately integrated to optimize the feature space of each node. Finally, we train corresponding classifiers to distinguish different activity groups and to classify a new unseen sample into one of the leaf-nodes in a top-down fashion to predict its activity label. To evaluate the performance of the proposed framework and feature selection methods, we conduct extensive comparative experiments on publicly available datasets and analyze the model complexity. Experimental results show that the proposed method reduces the dimensionality of original feature space and contributes to enhancement of the overall recognition accuracy. In addition, for feature selection, returning multiple activity-specific feature subsets generally outperforms the case of returning a common subset of features for all activities.

## 1. Introduction

With the rapid development of network communication technology and the miniature and high portability of various sensing units, a variety of applications [1,2,3], ranging from activity reminders and anomaly detection to fall detection, rehabilitation instruction, and wellness evaluation, are constantly emerging in the fields of ambient assisted living systems, smart home, smart building, healthcare, industry, security, etc. Among these meaningful applications, activity recognition plays a central role in better understanding the relationship between humans and their surroundings, where it essentially bridges the gap between the low-level streaming sensor data and high-level demand-oriented applications [4,5]. Due to the inherent nature of human behavior, however, human activities are characteristically associated with uncertainty, diversity, concurrency, and overlap. Specifically, there exists *inter-subject variation* in performing an activity, where different people largely perform an activity in a different way. Furthermore, even an individual can perform the same activity differently at different places and times, which is known as the case of *intra-subject variation*. For example, an individual may walk with different human body movement patterns and different walking speeds, and one can prepare coffee drinks in a different way of brewing and blending coffee recipes. Besides, an individual may execute concurrent and interleaved activities in other scenarios. One typical example in the smart home application is that one can cook dinner while watching TV. What is even more challenging is that there are activities that share very similar sensor readings, which easily weakens the discriminant ability of the trained data-driven activity classification models and inevitably results in a poor recognition performance. Consequently, real-world human activity recognition is a challenging but meaningful research topic that has attracted the attention of researchers from different fields [4,5].

For real-world human activity recognition, researchers have explored a variety of sensing technologies for a higher recognition performance and better adaptation to different application scenarios. Generally, existing activity recognition methods can be broadly categorized into three groups according to the underlying sensing technology used: vision-based methods, environmental sensor-based methods, and wearable sensor-based methods [6,7,8]. Vision-based methods basically employ a video or camera to capture a series of images and then utilize these to recognize different activities [9]. One typical example is Kinect for Xbox 360 that can sense human body motion in real-time. Although vision-based methods achieve a satisfactory performance in many cases, the use of a camera is not welcome and even not allowed in many scenarios, especially when the privacy issue is considered. Therefore, vision-based methods are often used for games, security, and safety surveillance. Besides, since cameras often suffer from background change, illumination variations, environmental noise, and ambient occlusion, the actual use of vision-based methods is further limited. Another important factor that prevents the wide use of vision-based methods is the high cost of hardware purchase and deployment. For environmental sensor-based methods, they are usually used in indoor scenarios for activity recognition and behavior analysis [8,10,11]. Assuming the underlying relationship between objects and activities, they usually place or embed sensing units on household objects and then infer ongoing human activities by recording and analyzing the interactions between an individual and objects. For instance, if we observe continuous sensor firings from a bed, someone is probably sleeping, and we probably infer that someone is leaving home, if the reed contact is triggered. Such a scheme involves a complex process to set up and maintain the system and has a low degree of portability. In addition, it is not trivial to deploy environmental sensors without causing much inconvenience to residents and bringing much change to the house layout. Unlike the two previous methods, benefiting from the miniature and flexibility of sensing units, wearable sensor-based methods perform activity recognition by allowing an individual to wear or carry mobile devices. Generally, wearable sensor-based methods have a wide range of applications and are suitable for indoor and outdoor scenarios and even extreme conditions, such as deep sea and outer space [12,13]. Besides, wearable devices can be worn on different parts of the human body, such as the arm, leg, wrist, waist, and neck, and an individual can wear multiple sensing devices simultaneously [14,15,16]. Among these wearable devices, the wide adoption of smartphones and strong user engagement provide us with an economic and efficient way for raw streaming sensor data collection and activity recognition [17]. Undoubtedly, these further increase the power and flexibility of wearable sensor-based methods.

In wearable sensor-based methods, the performance of a trained activity recognizer is largely determined by the choice of classification model and selection of the corresponding feature subset. Accordingly, researchers have conducted considerable work in exploring effective and efficient classification models. Commonly used classification models include naïve bayes, *k*-nearest-neighbor, neural network, decision tree, support vector machine, random forest, hidden Markov model, and conditional random field [18,19]. They have advanced this area and obtained satisfactory performances in recognizing highly different human activities, while most of the existing work, to the best of our knowledge, adopts flat classifiers that treat all the activities to be predicted at the same level. Flat classifiers try to construct a classifier to distinguish all the pre-defined activities in a single step at the cost of ignoring the hierarchical relationship among activities. On the other hand, there are human activities that generate quite similar sensor readings, which makes it difficult to discriminate such activities. One solution to this situation is to design a finer-grained classification model to gradually classify similar activities. In terms of feature engineering, most of the existing work aims at extracting a wealth of time-domain and frequency-domain features to better characterize the raw sensor signal. These methods tend to include irrelevant and redundant features, which leads to a classifier with a poor generalization capability and lowers the recognition accuracy. One feasible way to mitigate this problem is to identify a subset of discriminant features using feature selection methods [20]. Currently, there are a wealth of feature selection methods available and researchers have explored many of them to build an activity recognizer, but most of them seek to find a common subset of features for the pre-defined activities of interest and may fail to reflect the unique characteristics of each specific activity. On the contrary, a unique subset is likely to be associated with each activity.

Overall, although existing work has achieved great progresses, there are points that need to be further improved, especially in relation to the construction of activity classification models and the selection of informative features. To better discriminate similar activities and utilize informative features, this study proposes the construction of a hierarchical activity recognition framework. Specifically, according to the characteristics of human activities, the proposed framework, rather than classifying all the activities simultaneously, recognizes activities in a hierarchical multi-stage way. From the view of machine learning theory, this *coarse-to-fine* strategy enables a classifier to emphasize the local data structure and to learn a better decision boundary between objects with similar properties. Furthermore, to filter out irrelevant and redundant features, a feature selection process is integrated into the framework to optimize the activity recognition model. This enables us to obtain a robust activity recognition model that can better classify different activities and even similar activities. The main contributions of this study are itemized as follows:

- We propose the construction of a hierarchical activity recognition framework. According to the characteristics of human activities, predefined activities of interest can be organized into a hierarchical structure. Once the hierarchical model is trained, the activity label of a new test sample can then be predicted in a top-down fashion. This helps construct a finer-grained classifier to better discriminate similar activities.
- We propose two different feature selection methods that are embedded into the hierarchical framework. One is class independent feature selection that selects one common subset of features, and the other is class dependent feature selection that returns multiple subsets of features. This helps optimize the selection of informative features and enhance the generalization capability of an activity recognizer.
- We implemented and evaluated the proposed framework and feature selectors. Extensive experimental results show the superiority of the proposed method in reducing the feature dimensionality and improving the activity recognition accuracy.

The rest of this paper is organized as follows. In Section 2, we discuss related work about human activity recognition in terms of underlying sensing technology, a classification model, and feature engineering. Section 3 details the proposed hierarchical framework, including how to train the corresponding activity recognizer and how to use it for activity recognition. In Section 4, we introduce the two proposed feature selection methods and show how to integrate the feature selection process into the hierarchical framework. Section 5 describes the experimental settings and presents the experimental results and analyses. Section 6 concludes this study and points out directions for future research.

## 2. Related Work

To better understand human behavior and serve human-centric applications, recent years have witnessed considerable work in exploring various sensing technologies and in proposing a wealth of classification models in order to pursue a higher recognition accuracy. It has been shown that different types of sensors have their own advantages in recognizing specific human activities for different application scenarios. Nowadays, there are plenty of sensors available for use, ranging from vision-based sensors and environmental sensors to wearable sensors [4,6,21]. As we discussed in the previous section, compared with the case of vision-based and environmental sensor-based methods, wearable sensor-based methods have the advantages of a low cost, high degree of portability, and wide range of application scenarios, and they are proven to be effective and gaining popularity. Commonly used wearable sensors include accelerometer, gyroscope, RFID, GPS, magnetometer, electrocardiograph, light, temperature, and humidity sensors. The main purpose of wearable sensors is to generate and record time-series sensor readings when they are worn on different parts of the human body or put into the pockets. Our task is to correctly associate raw sensor data with corresponding activity labels. Among these sensors, accelerometers are a favorable choice since they provide acceleration and velocity information that helps infer human activities. For example, Bao et al. [22] utilized five biaxial accelerometers of small sizes that were simultaneously attached to different parts of the human body (the right hip and four limb positions) to classify twenty daily activities. They recruited twenty volunteers and asked them to perform pre-defined activities in a natural way for experimental sensor data collection. Afterwards, they extracted a variety of time-domain and frequency-domain features from the raw signals with a sliding-window of fixed length, and then constructed a classifier to recognize twenty activities. In their experiments, they evaluated the performance of three different classifiers and the obtained maximum accuracy reached 84.0%. Tapia et al. [23] used five tri-axial accelerometers and a heart rate monitor to recognize thirty physical gymnasium activities and associated activity intensities. These devices were worn on the right arm, right leg, and waist of an individual. Finally, their system obtained recognition accuracies of 94.6% and 56.3% for the subject-dependent and subject-independent cases, respectively. This kind of scheme typically involves complex system configuration and may prevent users from long-term use.

To mitigate the inconvenience caused by carrying multiple wearable devices simultaneously, there are researches exploring the possibility of using one single device. For instance, Khan et al. [14] used a single triaxial accelerometer that was attached to the chest of an individual to recognize fifteen activities in a natural setting. Experimental results showed they achieved a satisfactory performance. Ravi et al. [24] used an accelerometer mounted onto the pelvic region of an individual to recognize eight activities (standing, walking, running, upstairs, and downstairs, sitting, vacuuming, and brushing teeth). They then proposed the construction of a metalevel classifier and achieved a better performance in comparison with base-level classifiers. One major drawback of a single accelerometer is that they easily fail to discriminate similar activities. Besides an accelerometer, RFID technology is widely used, and it also helps to recognize human activities [25]. For example, Kim et al. [26] constructed an indoor healthcare monitoring system to track the elderly who wore RFID tags. Generally speaking, RFID is typically used for mobility modeling, location tracking, and trajectory prediction.

In addition, there are studies that have explored the use of heterogeneous sensing units to enrich the feature set and to better characterize human activity. For example, Huynh et al. [27] built a wearable fall detection system with an accelerometer and a gyroscope, and designed a threshold-based activity recognition and fall detection algorithm. The used device was placed on the chest center to classify different daily activities such as standing, walking, sitting, running, and four fall schemes (forward, backward, right, and left sideway). Lee et al. [28] used a digital compass, a biaxial accelerometer, and a gyroscope to track a user and to recognize standing, sitting, and walking activities. Furthermore, the increasing power of smartphones, often embedded with an accelerometer, gyroscope, light, and GPS, provides an alternative method of human activity recognition. The use of smartphones releases users from carrying other devices and it has a relatively low intrusiveness and high adherence. Accordingly, there are studies on activity recognition using a smartphone. For example, Chiang et al. [29] used an accelerometer and GPS embedded smartphone to classify nine activities for self-wellness management. Stisen et al. [19] systematically investigated the power of smartphones in activity recognition and conducted experiments with nine users to evaluate the effect of mobile sensing heterogeneities.

Besides exploring a variety of sensing units, researchers have also utilized a great number of models to associate the streaming sensor data with corresponding activity labels. We can roughly categorize existing methods into two groups: knowledge-driven approaches and data-driven approaches [4]. Knowledge-driven approaches typically work with an abstract model of domain knowledge and do not require massive data for model training [30]. Also, the knowledge can be used across applications. Besides, knowledge-driven approaches are easy to interpret and can work with noise and incomplete data. Ontology modelling, logical modelling, and evidential theory are three representative techniques. For example, Chen et al. [31] applied logical knowledge and logical reasoning for explicit context and activity modelling and representation to recognize activities. Liao et al. [32] explored the use of evidential theory to infer the occurrence of an activity. One major limitation of knowledge-driven approaches is that users are required to provide the model domain knowledge and that it would be rather difficult to define the specification of an activity for a new domain.

In contrast, data driven approaches classify activities by first collecting a great amount of data, then training a classification model to build the relationship between raw sensor data and activity labels, and finally making predictions with the established classifier. Furthermore, according to the availability of data labels, we can categorize them into three groups: supervised learning methods with activity labels available, unsupervised learning methods without activity labels, and semi-supervised learning methods that can use labelled and unlabeled data. For the unsupervised learning scenario, there is no labelled data available and such methods try to find the inherent data structure, where pattern mining algorithms and clustering algorithms are commonly used. One typical application of these methods is to discover new activities that have not been observed by users. Hence, they can be used in the initial stage of activity recognition where there are less labelled data. To achieve a high accuracy, supervised learning with an explicit training phase is much more widely used. Researchers have proposed a large number of supervised models that are mainly grouped into discriminative models (e.g., decision tree, conditional random fields, neural networks, and support vector machine), generative models (e.g., naïve bayes, hidden Markov blanket, and Bayesian network), and ensemble models (e.g., random forest, stacking, and boosting). For example, Javier Ordónez et al. [8] used artificial neural networks and a support vector machine within the framework of a hidden Markov model to take advantage of both a discriminant model and a generative model. Experimental results show a better recognition performance. As for the working scheme, most of these studies work on a large number of flat organized activity labels and often fail to explore information about the hierarchical relationship among activities.

Besides exploring powerful classification models, feature engineering is another important factor that largely determines the recognition performance. In practice, users tend to extract an over-complete set of features from the raw sensor data to better characterize the original signal. However, the obtained feature space probably contains redundant and irrelevant features that will deteriorate the performance of an activity recognizer. Consequently, there are studies that propose the utilization of feature selection methods to optimize feature space. For example, Gupta et al. [20] performed an Relief-F and sequential forward floating search to identify informative features. They evaluated the effectiveness of the feature selection method by conducting comparative studies on seven subjects with leave-one-person-out cross validation. Currently, there are many feature selection methods available for activity recognition, but most of them return a common subset of features for the pre-defined activities and thus fail to reflect the difference of each activity. Accordingly, this study aims to explore the use of a class dependent feature selection method and its combination with a hierarchical classification framework in human activity recognition.

As for the hierarchical framework, there are previous studies from related fields that propose to recognize human activities in a hierarchical structure. For example, under the assumption that each activity consists of a sequence of actions, van Kasteren et al. [33] proposed a two-layer hierarchical hidden Markov model for human activity recognition, where the bottom layer indicates the action clusters and the top layer indicates the activities of interest. This scheme automatically finds the action clusters, and it is not necessary for users to annotate sensor data with action labels. Finally, they compared their approach with two non-hierarchical temporal models on real-world datasets. Experimental results demonstrate the superiority over its competitors. For wearable sensor-based activity recognition, the cost of network communication, the requirement of real-time prediction, and the portability of wearable devices are key elements to the successful deployment of a practical system. To enable this, Wang et al. [34] proposed a hierarchical model to recognize human activities using a wireless body sensor network. Their model works in two consecutive steps. Specifically, the first step takes the acceleration data as the input and uses a lightweight template-match algorithm to recognize human gestures at each sensor node. In the second step, the output of the first step and other sensor readings are transmitted to a centralized device from the sensor nodes, and an Emerging Pattern-based algorithm recognizes the on-going activity in real-time. Experimental results show that this scheme not only achieves a better recognition performance, but also saves the network communication cost. For the above two studies, from the view of the prediction procedure, the activity label of a test sample is obtained in a down-top fashion, where the most specific activity label is first associated with the test sensor data and then a higher-level activity label is inferred with the predicted labels of the previous level. Without confusion, we call this scheme the *fine-to-coarse* strategy. This is quite different from our proposed hierarchical framework, where the label of a new test sample is predicted in a top-down fashion. This *coarse-to-fine* strategy enables a classifier to emphasize the local data structure and to learn a better decision boundary between objects with similar properties. In addition, we make no assumption that each activity consists of a sequence of actions, where complex temporal relationships are involved, and it is usually not trivial to accurately capture such temporal information. Besides, another significant difference between our work and the above two studies is that feature selection is integrated into the proposed hierarchical framework. Specifically, we propose two different types of feature selection methods and compare their performance in enhancing the generalization capability of an activity recognizer, while the two comparative studies used all the extracted features and failed to optimize the feature space. Accordingly, in this study, we propose integrating feature selection into the hierarchical activity recognition framework to achieve a robust activity recognizer that can better classify different activities and even similar activities.

## 3. Hierarchical Activity Recognition Framework

**Figure 1:**
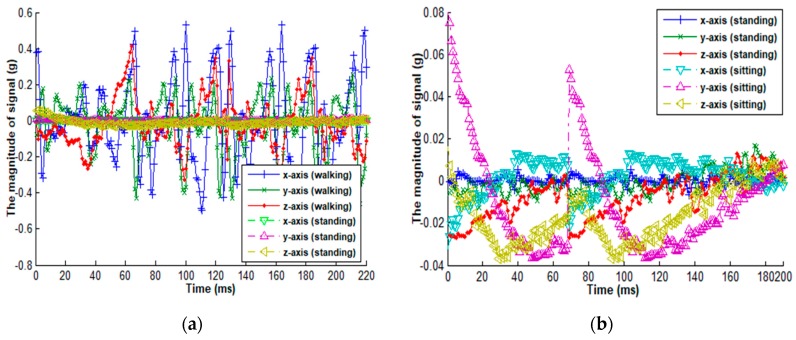
Comparison of the magnitude of a tri-accelerometer among three different activities. The accelerometer has sensor readings from three axes, i.e., x-axis, y-axis, and z-axis. (**a**) Comparison of walking and standing; (**b**) Comparison of standing and sitting.

Due to the inherent nature of human behavior, human activities are typically characterized by diversity, uncertainty, concurrency, and overlap. For wearable sensor-based methods, some activities can cause the used sensors to generate quite similar sensor readings, which further poses great challenges to the activity recognition model. For example, Figure 1 presents the signal magnitudes of an accelerometer associated with walking, standing, and sitting activities, from which we can observe that there is a great difference in sensor readings between walking and standing compared to the difference between standing and sitting. Therefore, training a classifier to distinguish walking, standing, and sitting activities probably fails to achieve a high performance, largely because this model would pay much more attention to walking and its non-walking activities and less attention to distinguishing standing and sitting. One feasible solution is to categorize human activities into static activity and dynamic activity according to human movement states. The former usually has small sensor readings, and the latter has sensor readings with a large amount of variance. Since some activities have quite different sensor readings and some activities have similar sensor readings, we can divide the predefined activities of interest into multiple sub-groups and further recognize activities within each sub-group.

Different from a flat classification model that works on a number of flat organized classes, the hierarchical activity recognition framework organizes the predefined activities of interest into a hierarchical tree according to the characteristics of human activities. We can then train an activity recognizer under the framework and predict the activity label of an unseen sample. In practical use, research on activity recognition is often oriented to specific applications and it is essentially difficult to consider all activities due to the complexity of human activities and the different granularities in defining activities. For example, pouring water is an activity to be predicted in one application, while it could be a component of making coffee in another application. Therefore, to explore the effectiveness of the proposed method and facilitate the discussion, this study considers six typical human activities (standing, sitting, lying, walking, upstairs, and downstairs) as an illustrative example.

**Figure 2:**
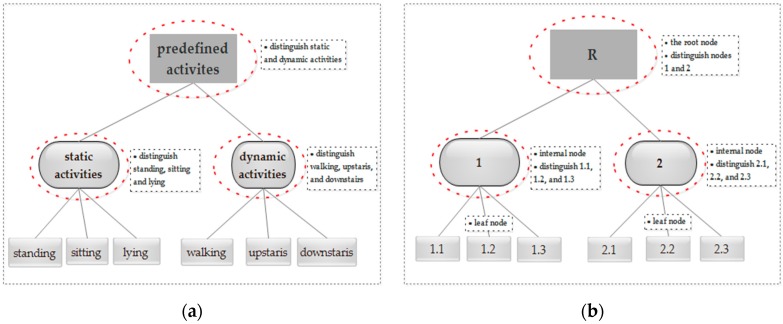
The proposed hierarchical activity recognition framework. The framework essentially organizes the predefined activities of interest into a hierarchical tree that indicates the relationship among different activities. (**a**) An example of a hierarchical activity recognizer; (**b**) Abstract form of the corresponding hierarchical structure.

These six basic human activities are also widely used in many other research studies [4,10,11]. According to the movement state, we can group standing, sitting, and lying into static activity, and group walking, going upstairs, and going downstairs into dynamic activity, and we can then recognize activities within each group. This helps to find a better decision boundary and enables the classifier to better discriminate similar activities. Figure 2 presents the proposed hierarchical activity recognition framework, which is basically a tree structure. Each internal node of the tree represents a group of activities with similar characteristics, each leaf node indicates a specific activity label, and the root node contains all the predefined activities of interest. Specifically, Figure 2a presents the hierarchical structure of the six basic activities, and Figure 2b is the abstract form of the corresponding hierarchical structure.

To clarify the procedure of how to train a hierarchical activity recognizer, how to integrate feature selection into the framework, and how to use it for activity recognition, we first introduce some notation.

*Tr* is a training set with *m* instances, *n* features, and one target variable *C*; *C* is the set of activity labels associated with the predefined activities of interest, and |*C*| equals the number of activities; *F* = {*F*_1_, *F*_2_, …, *F_n_*} is the original feature space of *Data*, and *F_i_* is the *i*-th feature of *F* (1 ≤ *i* ≤ *n*). For a node *nd*, we take the corresponding label as *C_nd_*. Then, we can define the ancestors, parents, children, descendants, and siblings of *nd* and their labels. Corresponding notation is given in Table 1.

### 3.1. Hierarchical Activity Recognizer Training

To fully utilize the hierarchical structure and avoid the inconsistency of activity label prediction, local classifier approaches are an option for training the activity recognizer, where they build a classifier for each non-leaf node, i.e., the nodes enclosed in dashed-line ovals. Specifically, for each non-leaf node *nd*, its training set *Tr*(*nd*) comes from its child nodes, and the number of children |↓(*nd*)| determines whether it is a binary or multi-class classification problem to handle for *nd*. Correspondingly, the task of *nd* is to distinguish its children from each other. For example, for the “root” node, its training set consists of the samples from its two children, i.e., the “static activity” node and “dynamic activity” node, and thus it is a binary classification problem; the training set of the “static activity” node consists of the samples from its three children, i.e., “standing”, “sitting”, and “lying”, and thus it is a three-class classification problem. The above analyses show that the hierarchical framework should build three classifiers. In comparison with the flat classifier, the proposed scheme can reduce the classifier complexity and mitigate the class imbalance problem. Particularly, we need to create a meta-class for the non-leaf nodes in implementing the structure. That is, in Figure 2b, classes associated with nodes R, 1, and 2 are meta-classes and we can name it ourselves as long as there is no conflict. The number of classes of a meta-class for a node *nd* equals the number of children of *nd*. Accordingly, we can obtain the training set of *nd* by utilizing the label relationship (e.g., ↑(*node* 1) is R, and ⇑(*node* 1.1) is R) and set the positive and negative class using ⌐(↓(*nd*)). In addition, although the depth of the example hierarchical framework is two, the depth of the framework largely depends on the specific applications.

### 3.2. Incorporating Feature Selection into the Framework

Feature selection methods have shown their power in improving the generalization capability and helping facilitate interpretation, which motivates us to integrate the feature selection process into the hierarchical activity recognizer towards a better performance. That is, feature selection is performed on each of the non-leaf nodes. Specifically, for non-leaf node *nd*, a feature selector is applied to its training set *Tr*(*nd*) and returns an optimized feature subset *S_nd_* associated with *nd*. Finally, an activity recognizer is trained with *Tr*(*nd*) and *S_nd_*. Algorithm 1 presents the pseudo-code of how to construct a hierarchical activity recognition model.


**Algorithm 1. The Pseudo-code of the Hierarchical Activity Recognition Method**
Inputs:   (1) dataset with activity labels   (2) the hierarchical tree *T* associated with the activity labelsOutput:   (1) a set of classifiers and feature subsets1. *cls_fs* = {}; //a set used for storing outputs2. **For** each non-leaf node *nd*
**do**  (2.1) search the child nodes ↓(*nd*) of *nd* with *T*;  (2.2) obtain a training set *Tr*(*nd*) using ↓(*nd*);  (2.3) do feature selection on *Tr*(*nd*), and get *S_nd_*;  (2.4) construct a classifier *cls_nd_* using *Tr*(*nd*) and *S_nd_*;  (2.5) *cls_fs*.add(< *cls_nd_*, *S_nd_*>); // add it to *cls_fs*3. **return**
*cls_fs*

### 3.3. Predicting with the Hierarchical Framework

To predict the label of an unseen sample, a top-down fashion is used to gradually predict its most specific activity label. Specifically, it works in the following scheme: (1) it first predicts the first level class; (2) it uses the predicted label to narrow the choices of classes; (3) it predicts the next-level class within the obtained choices; and (4) it repeats the above two steps until it gives the most specific prediction. That is, it reaches the leaf-node, which represents the activity label of interest. Algorithm 2 presents the pseudo-code of how to make predictions using the hierarchical model, in which feature subset projection is conducted on each non-leaf node and the maximal likelihood estimation is utilized to decide the class of a sample. It is noteworthy that the hierarchical model has a similar expression form to that of a decision tree, but they have essentially different meanings and have different training and testing procedures. First, the non-leaf node of a decision tree represents a partition of the feature space, while each of the non-leaf nodes of the hierarchical model indicates a classifier and often contains more than one feature. Second, in model training, the decision tree seeks the best partition feature in a top-down fashion according to a rule and generally generates a sub-tree, while the hierarchical model conducts feature selection and model training on each non-leaf node. Third, in prediction, the hierarchical model makes the prediction according to the prediction results and confidence along the top-down path, while the decision tree makes the prediction according to the comparison results between the test sample and the threshold value of an internal node.


**Algorithm 2. The Pseudo-code of Activity Recognition Using the Hierarchical Framework**
Inputs:   (1) a test sample *x*   (2) a set of classifiers and feature subsets *cls_fs*Output:   (1) the activity label of *x*1. set the root node as current node *nd*; //initialization2. obtain the number of children of *nd*, and note it as |↓(*nd*)|;3. project *x* over *S_nd_*, and use *cls_nd_* to get the next-level label *PL* and corresponding next-level node *pnd*, in which 1 ≤ *pnd* ≤ |↓(*nd*)| and *PL* corresponds to the maximal probability output of *cls_nd_*4. if *is leaf*_*node*(*k*) // conditional statement  (4.1) **if TRUE do**  **return**
*PL* as the predicted activity label;  (4.2) **if FALSE do**  set node *pnd* as current node, and go to step 2;

## 4. Feature Selection Methods

In human activity recognition, most of the current studies aim to extract new features and fail to optimize the used feature space, which leads to a classifier with a poor generalization capability. For the hierarchical framework, we propose two different feature selection methods to optimize the feature space for each non-leaf node: class independent feature selection and class dependent feature selection. This section mainly introduces how to integrate the two feature selection methods into the hierarchical framework.

### 4.1. Class Independent Feature Selection

The class independent feature selection method takes the training set as the input and applies a feature selection algorithm to identify a common subset of features. For the multi-class problem, a common feature subset probably fails to characterize each activity class. For example, one task is to recognize three different activities: *A*_1_, *A*_2_, and *A*_3_. Suppose one is performing *A*_1_ if and only if the value of feature *F*_1_ is less than a threshold; *A*_2_ is performed if and only if the value of feature *F*_2_ is above a certain limit; *A*_3_ is performed if and only if the value of feature *F*_3_ is below a certain limit; and all the other features are irrelevant to this recognition task. Afterwards, if a class independent feature selection algorithm is applied, it would return a feature subset that consists of *F*_1_, *F*_2_, and *F*_3_. Consequently, if a test sample is associated with *A*_1_, then *F*_2_ and *F*_3_ are noisy features to the classifier.

### 4.2. Class Dependent Feature Selection

**Figure 3:**
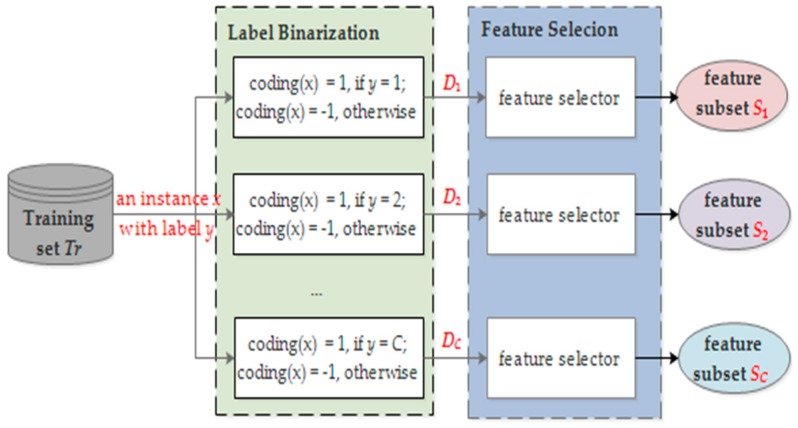
The proposed class dependent feature selection method. For a *C*-class problem, *C* feature subsets are finally returned, and they are probably different from each other.

In fact, different activities are generally represented by different subsets of features. Different from the class independent feature selection, class dependent feature selection finds an optimized feature subset for each activity and the obtained subsets are probably different from each other. The core idea of class dependent feature selection is to convert a *C*-class classification setting into *C* (*C* ≥ 3) two-class classification cases and conduct feature selection for each binary classification problem. Specifically, given a training set *D*, for the *k*-th (1≤ *k* ≤ *C*) classification problem, we obtain its training set *D_k_* by coding the label of an instance *x* with a label binarization operation: label *x* with +1 if its original label is class *k*; otherwise, label it with −1. Then, we obtain *C* training sets {*D*_1_, *D*_2_, …, *D_C_*} associated with the *C* binary classification problems. Afterwards, feature selection is applied to *D_k_* to obtain the optimal feature subset *S_k_*. In this step, class independent feature selection methods can be used. Finally, for a *C*-class classification problem, we obtain *C* feature subsets {*S*_1_, *S*_2_, …, *S_C_*}, each of which corresponds to a specific class. For the example in the previous sub-section, class dependent feature selection methods generate three feature subsets, i.e., {*F*_1_}, {*F*_2_}, and {*F*_3_}, and three classifiers. Since classifiers are trained with different feature subsets, there will be no noise for the classifiers and a better classification performance is expected. Figure 3 presents the scheme of class dependent feature selection, in which label binarization is performed to get *C* different training sets and returns *C* feature subsets {*S*_1_, *S*_2_, …*S_C_*}.

### 4.3. Hierarchical Activity Recognition with Class Dependent Feature Selection

**Figure 4:**
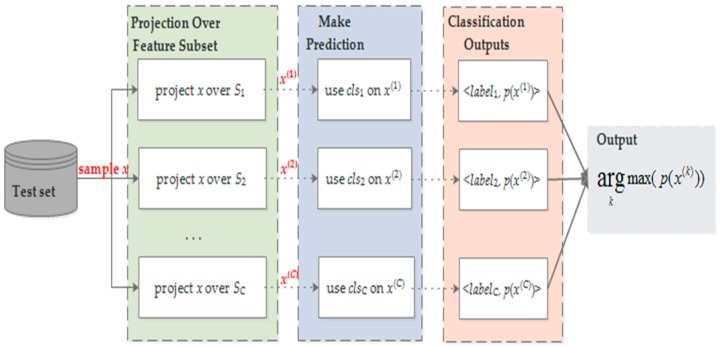
The scheme of classifying a test sample with the class dependent feature selection method. It first projects the test sample over different feature subsets, then uses the trained classifiers to make predictions, and finally determines the predicted label according to the maximal probability rule.

Since class independent feature selection methods return one feature subset, the procedure of model training and testing is similar to the case of without feature selection. As shown in Figure 4, for class dependent feature selection, however, multiple classifiers are to be trained and the principle of maximal likelihood estimation is used to make predictions. Specifically, for a non-leaf node, a classifier *cls_i_* is trained on *D_i_* using *S_i_*. Given a test sample *x*, we first project *x* over *S_i_* and get *x*^(*i*)^. Then, model *cls_i_* works on *x*^(*i*)^ and outputs its predicted label and probability estimation *p*(*x*^(*i*)^). With this, the label *L*(*x*) of *x* is determined by the maximal probability value.

*L*(*x*) = **max**_1 ≤ *i* ≤ *C*_ {*p*(*x*^(*i*)^)}, (1)

**Figure 5:**
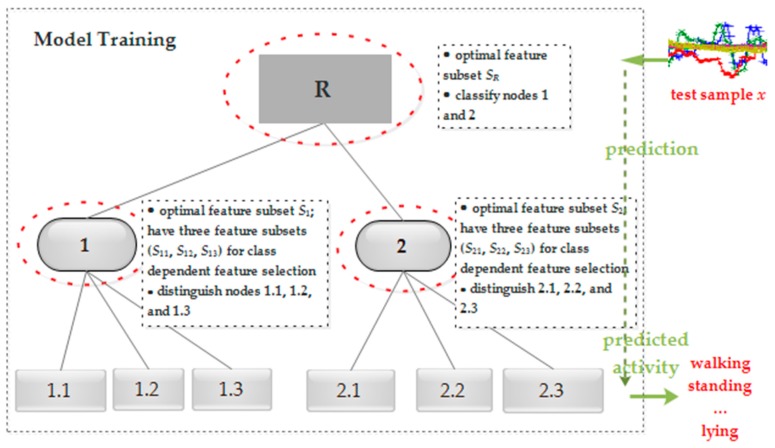
The proposed hierarchical activity recognition framework with feature selection integrated. In the phase of training, it selects the optimal feature subset and builds a classifier for each of the non-leaf nodes. In the phase of prediction, it adopts a top-down fashion to give the predicted label.

To better understand the hierarchical framework with feature selection, Figure 5 illustrates how to integrate feature selection into model training and how to classify an unseen sample in a top-down fashion. Specifically, to predict the activity label of a test sample *x*, the classifier of node R first projects *x* over its feature space *S_R_* and conducts the first-level classification, which outputs node 1 or node 2. During prediction, the maximal likelihood estimation is utilized to decide the class of a sample. If the prediction result is node 1, then the classifier of node 1 projects *x* over its feature space *S*_1_ and classifies the test sample into nodes 1.1, 1.2, or 1.3, which are leaf nodes and represent the finally predicted activity label; if node 2 is predicted, then the classifier of node 2 conducts further classification. This procedure returns the predicted label when a new sample comes. Afterwards, in the performance evaluation, the predicted labels are compared with corresponding true labels to measure the quality of an activity recognizer.

## 5. Experimental Setup and Results

To evaluate the effectiveness of the proposed method in wearable sensor-based activity recognition, we apply it to recognize six basic human activities. Besides, to evaluate the role of different types of sensing units in activity recognition, we consider the following three different configurations: (1) only using a tri-axial accelerometer; (2) only using a tri-axial gyroscope; and (3) using a tri-axial accelerometer and a tri-axial gyroscope. Such a setting enables us to evaluate the synergic effect of different sensing units. Our next task is to design an effective feature selection algorithm to improve the performance of an activity recognizer. For the illustration purpose, we call the flat classification model a non-hierarchical model. For the construction of a classifier, both the non-hierarchical model and hierarchical model can adopt class independent feature selection, as well as class dependent feature selection, to optimize the feature space. As we discussed, the proposed hierarchical activity recognition model is a general framework and can work with different feature selectors to build a hierarchical feature selection method. Considering that mutual information has the capacity to capture higher order statistics of data and non-linear relationships among features, an information theory-based feature selector, a fast correlation-based filter (FCBF), is integrated into the framework to eliminate irrelevant and redundant features [35]. Also, our preliminary results show FCBF can obtain a subset of features that are less redundant to each other and highly relevant to the target class. In addition, to evaluate the effectiveness of a feature selection method, a classifier is required to measure the goodness of the finally obtained feature subset. Herein, the widely used naïve bayes classifier is utilized due to its simplicity and effectiveness. Particularly, naïve bayes is sensitive to redundant features, which helps illustrate the necessity of feature selection.

**Figure 6:**
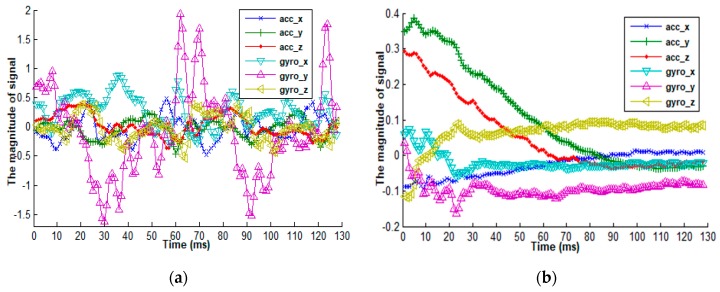
A comparison of the senor readings between a tri-accelerometer and a tri-gyroscope when an individual is performing an activity. (**a**) The situation of going upstairs; (**b**) The situation of sitting.

As for the wearable devices, in this study, a Samsung Galaxy S II smartphone equipped with a tri-axial accelerometer and a tri-gyroscope is used to collect raw sensor signals associated with different activities. The used smartphone, configured with a 1.2 GHz processor, a memory with 1 GB, and a Li-Ion battery with 1650 mA, can work continually for several hours, which satisfies the experimental requirements. The acceleration is obtained with the STMicroelectronics K3DH 3-axis accelerometer (measurement range ± 2 G, resolution = 0.0625, G is the gravitational constant) and the angular velocity is measured by the K3G gyroscope sensor (maximum range 8.7267, resolution = 0.0003). A gyroscope can measure the angular velocity that is generated by itself-rotation, and an accelerometer can obtain the magnitude and direction of a moving object. Figure 6 presents the sensor readings of a gyroscope and an accelerometer when an individual is going upstairs and sitting, from which we can view the significant difference between their signals. The x-axial represents time and the y-axis indicates the magnitude of different sensor signals. Specifically, the accelerometer has three axes (acc_x, acc_y, and acc_z) and the gyroscope has three axes (gyro_x, gyro_y, and gyro_z).

The experimental dataset was collected within a group of thirty volunteers aged between nineteen and forty-eight. These volunteers performed a protocol of activities with a smartphone attached to their waist. The activities of interest consist of three static activities (i.e., sitting, standing, lying) and three dynamic activities (i.e., walking, going upstairs, going downstairs). The sampling rate is 50 Hz and a sliding window of 2.56 s with 50% overlap between two adjacent segments is used to divide the streaming sensor data into segments [36]. That is, each window contains 128 sensor readings. Then, we extract 561 features from the window with 272 time-domain features and 289 frequency-domain features. Specifically, the features come from the raw sensor readings tAcc-XYZ and tGyto-XYZ that are generated by a tri-axial accelerometer and a tri-axial gyroscope, respectively. X, Y, and Z represent the three axes, and ‘t’ denotes the time-domain. Then, the acceleration signal is separated into the gravity acceleration signal (tGravityAcc-XYZ) and body acceleration signal (tBodyAcc-XYZ). For the body acceleration signal and angular velocity, Jerk signals (tBodyAccJerk-XYZ and tBodyGyroJerk-XYZ) are extracted. Subsequently, the signal magnitude of the acceleration and gyroscope data is obtained (tBodyAccMag, tGravityAccMag, tBodyAccJerkMag, tBodyGyroMag, tBodyGyroJerkMag). Besides time-domain information, frequency-domain information is extracted by applying a Fast Fourier Transform to the time-domain signals, which returns fBodyAcc-XYZ, fBodyAccJerk-XYZ, fBodyGyro-XYZ, fBodyAccJerkMag, fBodyGyroMag, and fBodyGyroJerkMag. ‘f’ denotes the frequency domain signals. After the above time-domain signals (starting with ‘t’) and frequency-domain signals (starting with ‘f’) are produced, a large set of features are extracted within the sliding window samples of these signals. A list of features is provided in Table 2.

Finally, the obtained dataset was randomly partitioned into two different sets, where data from 70% of the volunteers was used as training data and the rest as test data. Consequently, the experimental dataset includes 7352 training samples and 2947 test samples [37]. Table 3 presents the feature information about the dataset. The entry “gyro&acc” means “gyroscope and accelerometer”.

To measure the classification performance, a confusion matrix that consists of the predicted labels and true labels is used [38]. Table 4 presents a confusion matrix that is associated with three human activities: *A*1, *A*2, and *A*3. The entry “sum” of the last column and the last row indicates the sum of the corresponding row and column, respectively. For example, *NT*_1_ = *TP*_11_ + *FP*_21_ + *FP*_31_, *NI*_1_ = *TP*_11_ + *FP*_12_ + *FP*_13_, and *total* = *NI*_1_ + *NI*_2_ + *NI*_3_.

Based on the confusion matrix, we can define the accuracy and *F1* metrics and use them to compare the performance. A higher value of accuracy and *F1* indicates the superiority of the corresponding method.

*Accuracy* means the percentage of samples that are correctly classified for a *C*-class classification problem, as shown in Equation (2), where *total* equals the number of test samples.

(2) $$Accuracy=\frac{\sum_{i=1}^{C}TP_{ii}}{total}=\frac{\sum_{i=1}^{C}TP_{ii}}{\sum_{i=1}^{C}NI_{i}}=\frac{\sum_{i=1}^{C}TP_{ii}}{\sum_{i=1}^{C}NT_{i}}$$

*Precision* is the weighted average of the percentage of the correctly inferred labels for each class. *Precision* is calculated using Equation (3).

(3) $$Recall=\frac{1}{C}\sum_{i=1}^{C}\frac{TP_{ii}}{NT_{i}}$$

*Recall* means the weighted average of the fraction of the true labels that are correctly classified.

(4) $$Precision=\frac{1}{C}\sum_{i=1}^{C}\frac{TP_{ii}}{NI_{i}}$$

*F1* combines precision and recall into a single metric and it takes a value between 0 and 1.

(5) $$F1=\frac{2*precision*recall}{precision+recall}$$

We conducted experiments on a computer with a 3.2 GHz processor and 4 GB memory configuration. Table 5 and Table 6 present the experimental results of accuracy and *F1* for the hierarchical and non-hierarchical methods. The entry “no feature selection” indicates that we keep all the original features without using feature selection methods. The last row, “gyro&acc”, refers to the joint use of a gyroscope and accelerometer. Furthermore, to better understand the role of sensor fusion, feature selection, and a hierarchical framework, we performed further analyses from these three aspects.

### 5.1. Accelerometer vs. Gyroscope

**Figure 7:**
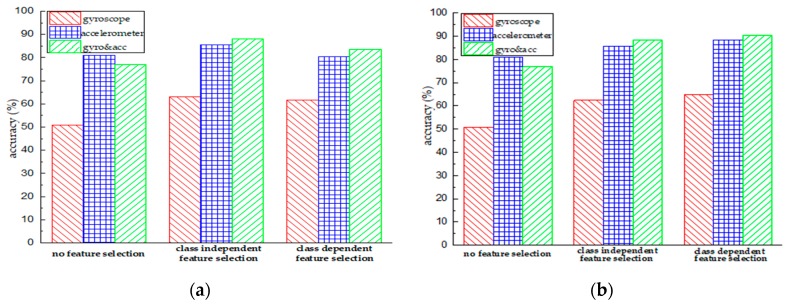
Performance comparison of using different sensing units in the case of no feature selection, class independent feature selection, and class dependent feature selection. (**a**) Performance obtained using the non-hierarchical model; (**b**) Performance obtained using the hierarchical model.

For wearable sensor-based human activity recognition, the tri-axial accelerometer and tri-axial gyroscope are two commonly used sensing units, but there are few comparative studies about their performance in activity recognition. Figure 7 shows the accuracy comparison of the accelerometer, gyroscope, and the joint use of the accelerometer and gyroscope. The x-axis represents different feature selection methods, and the y-axis indicates the classification accuracy. First, we observe that, compared with the gyroscope, the use of the accelerometer generally obtains a better recognition performance. For example, for the non-hierarchical method without feature selection, the use of the accelerometer achieves an accuracy of 81.07%, while the accuracy of using the gyroscope is 50.80%; for the hierarchical method, the accuracies associated with the gyroscope and accelerometer are 62.44% and 85.61%, respectively. For the hierarchical method with class dependent feature selection, the use of the accelerometer obtains an accuracy of 88.29% compared to 64.91% for the gyroscope. This indicates that if we only choose one sensing unit, the use of an accelerometer is superior to that of a gyroscope. Second, directly fusing sensor data from the gyroscope and accelerometer cannot guarantee the accuracy improvement in activity recognition. For example, for the non-hierarchical method, using the accelerometer obtains an accuracy of 81.07%, while the accuracy of using both the accelerometer and gyroscope decreases to 76.99%; similarly, for the hierarchical method, in comparison to the 76.86% accuracy that is obtained using the accelerometer and gyroscope, the accuracy of the accelerometer is 80.90%. This indicates that redundant and irrelevant information probably exists among the features extracted from the accelerometer and gyroscope data. Third, with the aid of a feature selection method, the joint use of the accelerometer and gyroscope consistently obtains a higher classification performance than that of just using the gyroscope or accelerometer. For example, for the non-hierarchical method, with the help of the class independent feature selection method, the use of both the accelerometer and gyroscope obtains an accuracy of 88.16%, which outperforms that of the accelerometer (an accuracy of 85.61%) and the gyroscope (an accuracy of 62.95%); after using the class dependent feature selection method, the accuracy of sensor data fusion is 83.61%, while the accuracies of using the accelerometer and gyroscope separately are 80.49% and 61.62%, respectively. This indicates that the accelerometer and gyroscope can provide complementary information to each other and that redundant information also exists among the extracted features.

### 5.2. Class Dependent vs. Class Independent Feature Selection

**Figure 8:**
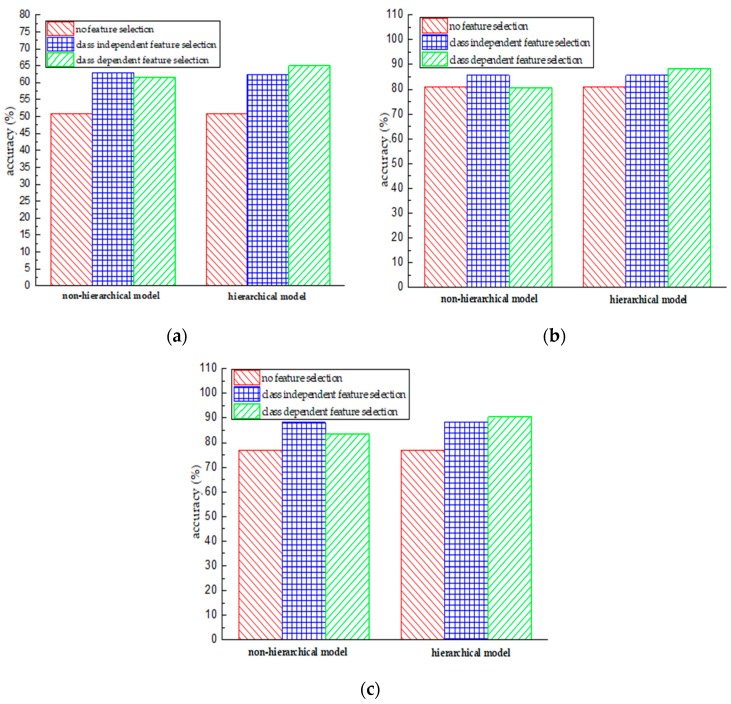
Performance comparison of using different feature selection methods in the case of the non-hierarchical model and hierarchical model. (**a**) Performance obtained using the gyroscope data; (**b**) Performance obtained using the accelerometer data; (**c**) Performance obtained using the accelerometer & gyroscope data.

The main differences between class dependent feature selection and class independent feature selection are the number of finally obtained features and the way of dealing with the dataset labels. Specifically, label binarization could increase the number of negative-class samples and exacerbate the issue of class imbalance, which leads to a poor generalization capacity of the corresponding classifier. Figure 8 presents the accuracy of the class independent feature selection method and class dependent feature selection method. The x-axis shows different classification models and the y-axis indicates the classification accuracy. We can observe that compared to the case of no feature selection, conducting feature selection indeed contributes to the enhancement of classification performance, no matter which sensing unit is used. This indicates that feature selection methods can eliminate redundant and irrelevant features from the original feature space, resulting in a better generalization capacity. Second, for the non-hierarchical model, the class independent feature selection method outperforms the class dependent feature selection method; but for the hierarchical model, the class dependent feature selection method works better. One explanation for this situation is that the use of the class dependent feature selection method in the non-hierarchical model exacerbates the problem of class imbalance. In contrast, the hierarchical model implicitly mitigates this problem by converting the original problem into smaller ones. For instance, for the activity “walking”, its ratio is 16.7% for the non-hierarchical model compared to 37.4% for the hierarchical model.

### 5.3. Hierarchy vs. Non-Hierarchy Model

**Figure 9:**
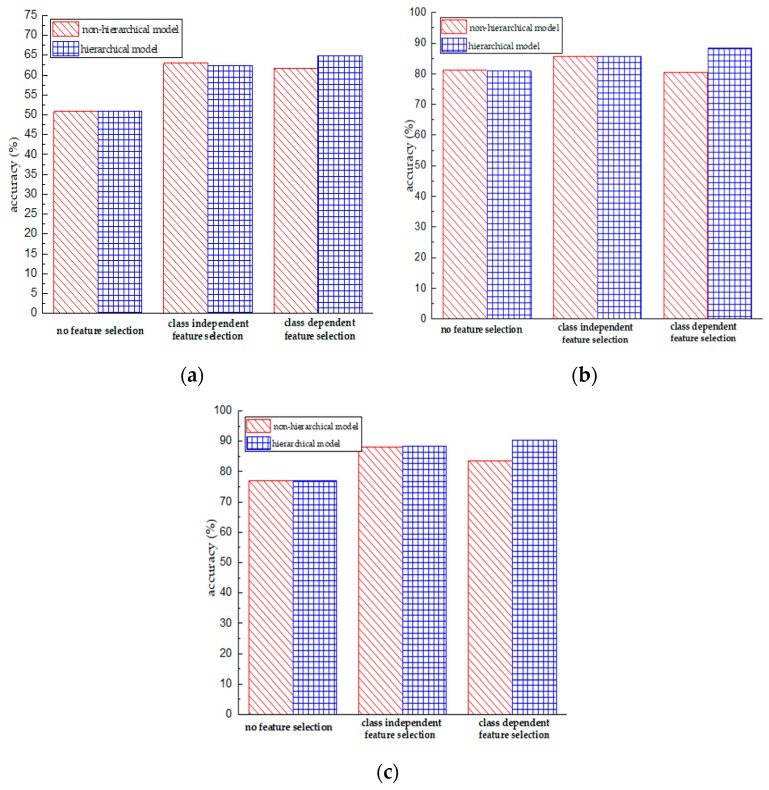
Performance comparison of using the non-hierarchical model and the hierarchical model in the case of no feature selection, class independent feature selection, and class dependent feature selection. (**a**) Performance obtained using the gyroscope data; (**b**) Performance obtained using the accelerometer data; (**c**) Performance obtained using the accelerometer & gyroscope data.

Compared with the non-hierarchical model, the hierarchical model utilizes the relationships among activities and recognizes activities in a hierarchical multi-stage way. This coarse-to-fine strategy enables a classifier to emphasize the local data structure and to learn a better decision boundary between objects with similar properties. Figure 9 presents the comparative results between hierarchical and non-hierarchical models. The *x*-axial represents different feature selection methods and the *y*-axis indicates the classification accuracy. We can observe that the hierarchical model outperforms the non-hierarchical model in the majority of cases. Specifically, they achieve a similar classification performance under the condition of using class independent feature selection or without feature selection; however, the hierarchical model performs better than the non-hierarchical model in the case of class dependent feature selection. According to the results of Table 5 and Table 6, we observe that the hierarchical model with class dependent feature selection achieves the best accuracy.

Overall, according to the above experimental results and analyses, we conclude that the use of the feature selection method and the fusion of different types of sensor data contribute to the enhancement of classification performance in human activity recognition. Also, compared with the non-hierarchical model, the hierarchical model is a better choice. Besides, to better understand the performance improvement brought about by the hierarchical model and feature selection method, we investigate the confusion matrix when both the accelerometer and gyroscope are used in the experiments. Table 7, Table 8 and Table 9 present the confusion matrices for the case of no feature selection in a non-hierarchical model, the case of non-hierarchical class dependent feature selection, and the case of hierarchical class dependent feature selection, respectively. The rows represent the true labels and the columns represent the predicted labels. The diagonal elements of the confusion matrix indicate the number of correctly predicted labels for each activity. According to the matrices, we observe that the feature selection method helps discriminate different motion states. For example, Table 7 recognizes 29 (4 + 7 + 15 + 3 = 29) dynamic activities as static activities mistakenly without using feature selection, while Table 8 reduces the number of errors to 21 (4 + 9 + 8 = 21) with a feature selector. Compared with the non-hierarchical model, we observe that the hierarchical model contributes to better distinguishing activities with similar patterns. For example, the non-hierarchical model mistakenly recognizes 41 “walking” samples as “upstairs/downstairs” samples, while the hierarchical model reduces this number of 26; the non-hierarchical model recognizes 267 “sitting” samples as “standing/lying” samples, and the hierarchical model only makes 92 wrong predictions.

### 5.4. Model Complexity

This section describes the time performance and compares the time costs of the hierarchical and non-hierarchical models. Since the hierarchical model builds a classifier for each non-leaf node, it inevitably causes extra time costs in making predictions compared to the case of non-hierarchical model. For the experiments in this study, the non-hierarchical model only builds a classifier, while the hierarchical model needs to build three classifiers. Table 10 presents the time cost comparison between the two models in predicting unseen samples. We can observe that the hierarchical model requires much more time than the non-hierarchical model. For example, for the case of no feature selection, the time costs are 0.1669 and 5.8641 s, respectively. Moreover, since feature selection reduces the dimensionality of data, the time complexity of a classifier with optimized feature space is highly reduced. For example, for the hierarchical model, the time costs for the cases of no-feature selection, class independent feature selection, and class dependent feature selection are 5.861, 3.0745, and 3.9127 s, respectively.

### 5.5. Complex Activity Recognition

Besides, we preliminarily investigate the performance of the proposed framework in recognizing relatively complex activities and conduct experiments on the publicly available dataset PAMAP2 [39]. The PAMAP2 dataset is collected by asking nine subjects to wear three inertial measurement units and a heart rate monitor and perform the predefined activities. The three measurement units are placed on the chest, on the dominant side’s ankle, and over the wrist of the dominant arm. After extracting a set of features from the raw sensor signals, we apply the proposed framework to this situation. The experimental dataset consists of 11,295 samples. To obtain an unbiased evaluation, ten-fold cross validation is used to randomly divide the dataset into ten folds, where one of the tens is used as a test set and the remaining nine folds are used as a training set. The final results are the average of the ten results, which is shown in Table 11. We observe that the hierarchical model achieves a better performance than its competitor.

## 6. Conclusions

With the rapid development of sensor technology and pervasive computing, wearable devices are widely used in the study of human behavior analysis and activity recognition, as well as in applications of smart homes, healthcare, sports, and rehabilitation, etc. Among these applications, activity recognition essentially bridges the gap between the low-level streaming sensor data and high-level demand-oriented applications. To further improve the performance of an activity recognizer and evaluate the role of different types of sensing units, this study proposes a hierarchical feature selection framework for human activity recognition and designs two different feature selection methods within the framework. Then, a specific activity recognizer is trained and compared with its competitors. Experimental results show that the proposed method contributes to enhancement of the overall recognition accuracy and that the joint use of multiple sensing units is superior to only using one sensing unit. In addition, returning multiple activity-specific feature subsets generally outperforms the case of returning a common subset of features for all activities.

For the future work, we plan to conduct further research along the following lines. First, we will explore the use of ontology and the clustering methods to automatically construct the relationships between activities. For example, rather than artificially grouping similar objects, clustering methods categorize similar objects into a cluster in a data-driven way, which has better expansibility in handling a large number of activities [40,41]. Second, how to utilize vision-based sensors, environmental sensors, and wearable sensors, and design an effective information fusion strategy, remains another research topic [42]. For example, the gyroscope is usually used in scenarios, such as navigation and mobile games, that need angle velocity; and except for an accelerometer, it is seldom used with other wearable sensors in human activity recognition. On the other hand, vision-based methods achieve a good performance in activity recognition. Therefore, we can investigate how to utilize two such heterogeneous information sources to build a roust and effective model.

## Figures and Tables

**Table 1:** Notation and corresponding meaning.

Symbol	Meaning	Remark
*Tr*	a dataset set	*m* instances, *n* features
*C*	the set of activity labels	the predefined activities
*↑*(*nd*)	the parent of *nd*	the parent node of *nd*
↓(*nd*)	the set of children of *nd*	the children node of *nd*
⇑(*nd*)	the set of ancestors of *nd*	the ancestor nodes of *nd*
⇓(*nd*)	the set of descendants of *nd*	the descendant nodes of *nd*
⌐(*nd*)	the set of siblings of *nd*	the sibling nodes of *nd*
*Tr*(*nd*)	the training set of *nd*	training set of *nd*
|↓(*nd*)|	the number of children of *nd*	binary or multi-class problems
*S_nd_*	an optimized feature subset of *nd*	obtained with feature selection

**Table 2:** A list of features for activity recognition.

Features	Features
mean value	standard deviation
maximum	minimum
median absolute deviation	signal energy
signal magnitude area	interquartile range
autoregression coefficients	bands energy
skewness of the frequency-domain signal	kurtosis of the frequency-domain signal
angle between two vectors	correlation coefficient between two signals
the frequency component with largest magnitude	weighted average of the frequency components

**Table 3:** Experimental dataset description.

Sensing Unit	The Number of Features		
	Time-Domain	Frequency-Domain	Total
gyroscope	106	105	211
accelerometer	164	184	348
gyro&acc	272	289	561

**Table 4:** Confusion matrix for the classification of three human activities.

		True Labels			
		*A*1	*A*2	*A*3	sum
Predicted labels	*A*1	*TP*_11_	*FP*_12_	*FP*_13_	*NI*_1_
	*A*2	*FP*_21_	*TP*_22_	*FP*_23_	*NI*_2_
	*A*3	*FP*_31_	*FP*_32_	*TP*_33_	*NI*_3_
	sum	*NT*_1_	*NT*_2_	*NT*_3_	*total*

**Table 5:** A comparison of accuracy between hierarchical and non-hierarchical methods.

Sensor	Non-Hierarchical Model			Hierarchical Model		
	No Feature Selection	Class Independent	Class Dependent	No Feature Selection	Class Independent	Class Dependent
gyroscope	50.80	62.95	61.62	50.87	62.44	64.91
accelerometer	81.07	85.61	80.49	80.90	85.61	88.29
gyro&acc	76.99	88.16	83.61	76.86	88.39	90.36

**Table 6:** A comparison of *F1* between hierarchical and non-hierarchical methods.

Sensor	Non-Hierarchical Model			Hierarchical Model		
	No Feature Selection	Class Independent	Class Dependent	No Feature Selection	Class Independent	Class Dependent
gyroscope	53.42	64.49	64.79	53.65	63.56	66.34
accelerometer	81.49	85.61	81.83	81.34	86.04	88.28
gyro&acc	78.03	88.09	84.66	77.90	88.72	90.34

**Table 7:** Confusion matrix for no feature selection of the non-hierarchical case.

	Walking	Upstairs	Downstairs	Sitting	Standing	Lying
**Walking**	416	9	80	0	4	0
**Upstairs**	38	451	83	7	15	3
**Downstairs**	42	11	257	0	0	0
**Sitting**	0	0	0	368	54	212
**Standing**	0	0	0	111	455	0
**Lying**	0	0	0	5	8	322

**Table 8:** Confusion matrix for non-hierarchical class dependent feature selection.

	Walking	Upstairs	Downstairs	Sitting	Standing	Lying
**Walking**	455	58	34	0	4	0
**Upstairs**	26	388	22	9	8	0
**Downstairs**	15	25	364	0	0	0
**Sitting**	0	0	0	215	15	0
**Standing**	0	0	0	262	505	0
**Lying**	0	0	0	5	0	537

**Table 9:** Confusion matrix for hierarchical class dependent feature selection.

	Walking	Upstairs	Downstairs	Sitting	Standing	Lying
**Walking**	470	27	18	0	1	0
**Upstairs**	8	440	35	4	17	1
**Downstairs**	18	4	367	0	1	0
**Sitting**	0	0	0	395	58	0
**Standing**	0	0	0	92	455	0
**Lying**	0	0	0	0	0	536

**Table 10:** Time cost comparison between hierarchical and non-hierarchical methods.

Methods	Non-Hierarchical Model (s)	Hierarchical Model (s)
No feature selection	0.1669	5.8641
Class independent feature selection	0.0047	3.0745
Class dependent feature selection	0.0001	3.9127

**Table 11:** Classification accuracy comparison of the PAMAP2 dataset.

Methods	Non-Hierarchical Model	Hierarchical Model
Class independent feature selection	0.9457	0.9928
Class dependent feature selection	0.9743	0.9796
